# Two new infraspecific taxa of *Verbascum delphicum* (Scrophulariaceae, Scrophularieae) from mainland Greece and the island of Evvia

**DOI:** 10.3897/phytokeys.74.10381

**Published:** 2016-11-10

**Authors:** Aris Zografidis

**Affiliations:** 1 Institute of Biosciences and Applications, National Centre for Scientific Research Demokritos, Aghia Paraskevi, Athens, Greece

**Keywords:** Verbascum, new subspecies, endangered taxon, Mt Parnitha

## Abstract

Verbascum delphicum Boiss. & Heldr. subsp. cervi Zografidis (Scrophulariaceae, Scrophularieae) is described as a subspecies new to science and illustrated. It is narrowly distributed in the Greek National Park of Mt Parnitha (Attica, Greece) with a very small population size. The new subspecies is a seldom-collected taxon, previously overlooked and misidentified as consubspecific with the autonymous subspecies, an endemic of the island of Evvia (Greece). Also described in this study is a new variety of subsp. delphicum from Mt Ochi of southern Evvia.

## Introduction

Comprising more than 360 species, *Verbascum* L. (Scrophulariaceae) is the largest genus of the predominantly northern temperate tribe Scrophularieae Dumort. (= Verbasceae Dumort.) and is chiefly represented by rosulate, biennial or perennial herbs with yellow-flowered, thyrsic or racemose inflorescences (Fischer 2004; Oxelman et al. 2005). Although the genus is widely distributed in Eurasia and North Africa, species diversity is mostly concentrated in a region encompassing Anatolia, Caucasus, northwestern Iran, the Levant and the southern Balkans (Murbeck 1939). In Greece, *Verbascum* is among the 10 most species-rich and the 10 most range-restricted-rich genera with a total of 76 species and an endemism rate of 32% (Dimopoulos et al. 2013).

*Verbascum* was extensively studied by the Swedish botanist Svante Murbeck who published two monographs on *Verbascum* and *Celsia* L. — now merged in *Verbascum* — and a series of two additional, supplementary and interpretative studies on the genus (Murbeck 1925; 1933; 1936; 1939). Since his era, several other species have been discovered and described, principally but not exclusively from Anatolia (AI-Hemaid 2001; Aytaç and Duman 2012; Bani et al. 2010; Çeçen et al. 2015; Davis 1951; 1952; Firat 2015; Greuter and Rechinger 1972; Huber-Morath 1939; 1949; 1955; 1960; 1965; 1967; 1971; 1973; 1974; 1975; 1976; 1979; 1981; 1983; Karavelioğullari 2015; Karavelioğullari et al. 2011; Karavelioğullari et al. 2004; Karavelioğulları et al. 2009; Karavelioğullari et al. 2008; Karavelioğullari et al. 2014; Kaynak et al. 2006; Parolly and Eren 2008; Parolly and Tan 2007; Ranjbar and Nouri 2015; Sotoodeh et al. 2015; Sotoodeh et al. 2016; Stefanova-Gateva 1979) and a modified classification system of “*informal, partly artificial groups*” has been utilized by Arthur Huber-Morath for the Turkish taxa (Huber-Morath 1978). Both taxonomies of Murbeck and of Huber-Morath are still widely cited and used today in studies of new species; however, it should be noted that a first assessment of molecular data suggests these schemes are non-natural and that the genus has potentially been subjected to taxonomic inflation (Ghahremaninejad et al. 2014).

*Verbascum delphicum* Boiss. & Heldr. was discovered by Theodor von Heldreich on Mt Dirfi of the island of Evvia (West Aegean Islands, Greece) and was subsequently jointly described by himself and Edmond Boissier in Boissier’s Diagnoses plantarum Orientalium novarum (Boissier 1856). The autonymous subspecies is a nemoral, short-lived perennial or biennial herb with often impressive, sizeable basal leaves. Knowing the taxon well from my excursions on the mountains of central and south Evvia I was surprised to find on Mt Parnitha (Attica, Sterea Ellas, mainland Greece) on May 2013 a small population of a closely related, yet distinct and undescribed as it appeared to be taxon. The population consisted of biennial plants with noticeably smaller and more or less differently shaped leaves than those of the insular *Verbascum delphicum*. Inspection of the mainland population in the past recent years and comparison with the insular populations revealed some additional deviating traits for the taxon on Mt Parnitha. Taking into consideration overall morphology and the common habitat and phenology of the two taxa, as well as the phytogeographic connection between Sterea Ellas and Ins. Evvia, I propose that collectively the differences indicate distinction at the subspecies rank.

The new subspecies Verbascum delphicum subsp. cervi Zografidis described and illustrated in this work was apparently overlooked and misidentified by Murbeck as consubspecific with subsp. delphicum based on material collected by Heldreich and Bassilios Tuntas from Mt Parnitha. The differences between the two subspecies are presented and discussed. A new, distinct variety of subsp. delphicum from Mt Ochi, south Evvia is also described.

## Methods

For the cultivated material current-year seeds were sown in early October (2013 & 2015), in small pots filled with Compo® Bio Anzucht- und Kräutererde substrate. Two-weeks old plants were individually transferred in plastic pots 7 cm of diameter and were transplanted every c. 8 weeks two additional times in successively larger plastic pots, 15 and 24 cm of diameter. The plants were kept outdoors, daily receiving 5 hours of direct sunlight on average, whereas the substrate was kept moist but not soggy.

Measurements were performed either with a common ruler or under a stereo-microscope (Zeiss, Stemi 2000-C) equipped with a camera and ImagePro software. Because the population size of the new subspecies is extremely small the measurements were either performed *in situ* without damaging the plants, or, when this was not feasible, only small plant-parts were collected for subsequent assessment under the stereo-microscope. In particular, two flower-clusters (glomerules) of the middle part of the inflorescence were removed from 20 individual plants i) at flowering and ii) at fruiting. Statistical significance was assessed with two-tailed T-tests for two independent means (*p*<0.01) and two-tailed Mann-Whitney U-tests (*p*<0.01).

## Taxonomy

### Verbascum delphicum Boiss. & Heldr. subsp. cervi

Taxon classificationPlantaeLamialesScrophulariaceae

Zografidis subsp. nov.

urn:lsid:ipni.org:names:77158533-1

**Figure 1.**
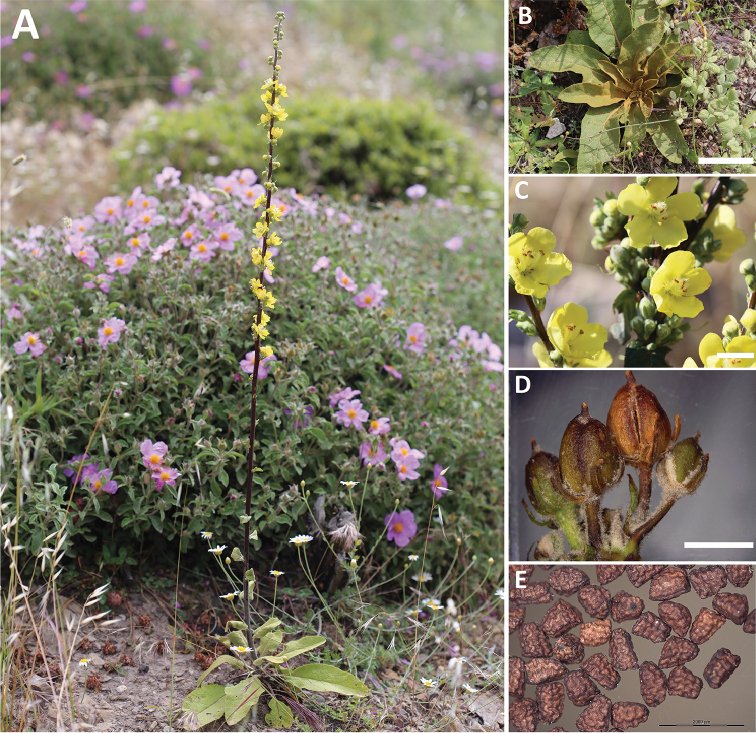
Verbascum delphicum subsp. cervi Zografidis: **A** habit **B** first year mature leaf-rosette, bar = 10 cm **C** partial inflorescence, bar = 1 cm **D** capsules, bar = 5 mm **D** seeds, bar = 2 mm.

**Figure 2.**
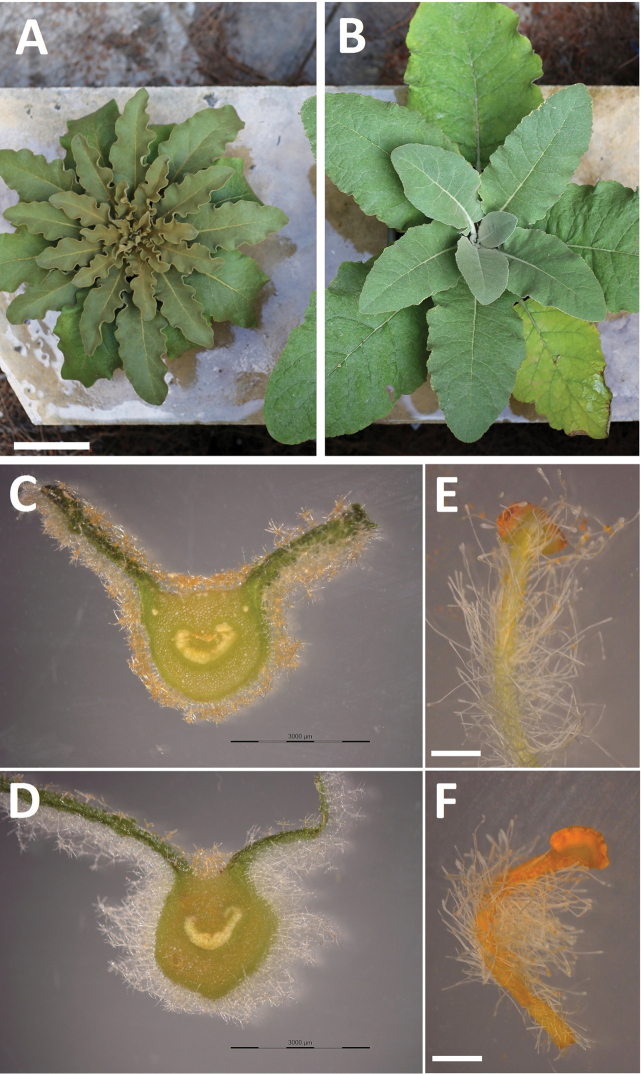
Comparative illustration of Verbascum delphicum subsp. cervi and subsp. delphicum differential traits: **A, C, E** subsp. cervi **B, D, F** subsp. delphicum **A, B** first-year mature rosette-leaves of cultivated plants, bar = 10 cm **C, D** cross section of leaves indicating the shorter and darker indumentum of subsp. cervi, bars = 3 mm **E, F** anterior stamens, bars = 1 mm;

Figures 1 , 2 ; Suppl. material 1

#### Diagnosis.

subsp. cervi can be distinguished from subsp. dephicum by a combination of the following characters: lamina of larger rosette-leaves 6–22 × 3–11 cm (vs. 16–40 × 11–24), 1.5–3.7 of length to width ratio (vs. 1.2–1.9), obtuse-cuneate at the base (vs. obtuse-truncate to cordate); indumentum of abaxial surface of rosette-leaves thinner; indumentum of adaxial surface of first-year mature rosette-leaves ± harsh (vs. soft); stamen filaments greenish-white (vs. orange).

#### Type.

GREECE. Attica: Mt Parnitha, 38°09'N, 23°43'E, limestone slope with *Abies cephalonica*, 1100 m, 22 June 2015, *A. Zografidis 109*. (Holotype: ATH, Isotype: ATHU)

#### Description.

Monocarpic, eglandular- and minutely glandular-hairy biennial herb — or rarely short-lived perennial bicarpic — producing a well-branched taproot and a basal leaf-rosette in the first year of vegetative growth, followed by the production of additional rosette-leaves (in the same rosette) and an erect, terete, leafy flowering-stem in the second year of vegetative and reproductive growth. Eglandular hairs dendritic, 0.2–0.8 mm of length, more or less covering the whole aerial part of the plant; glandular hairs minute, sparse, present on leaves, bracts, bracteols and calyx segments, visible by microscopy; fully developed first-season rosettes yellowish- to brownish- and harshly-tomentose above, grayish- or yellowish- to brownish-, and ± harshly-tomentose beneath; rosette-leaves few to several (up to 50 in cultivated specimens), petiolate; petiole 1–10 cm of length; lamina ovate-elliptic to oblanceolate, 1.5–3.7 of length to width ratio, obtuse-cuneate at the base, crenate, obtuse at apex; larger leaf laminas 6–22 × 3–11 cm; second year mature rosette-leaves and lower cauline-leaves similar but ± glabrescent on adaxial surface; middle cauline-leaves progressively smaller, shortly petiolate, obtuse at the base, obtuse or subacute at the apex; upper cauline-leaves, small, sessile, obtuse at the base, subacute at the apex; all cauline leaves alternate; stem 40–160 cm of height, green to reddish-black, glabrescent but ± persistently tomentose below; Inflorescence 25–60 cm of height, simple or sparingly branched at the base with short, sub-erect branches and then inflorescence narrowly pyramidal in outline; flowers arranged in clusters of pedicellate, compacted cymes (glomerules), ± crowded at least above, consisting of 3 – 12 flowers; bracts 3–7 × 2–4 mm, ovate-lanceolate, acute to acuminate, glabrescent; bracteoles present, similar to bracts but smaller; longer pedicels 3–8 mm of length, tomentose, ± glabrescent; calyx divided almost to the base into 5 lanceolate to lanceolate-linear, acute segments, 3.5–6 × 1.1–1.7 mm; abaxial surface of calyx tomentose, ± glabrescent; corolla rotate, flat, 1.6–3.6 cm of diameter, light yellow, often with purple marks on the throat, with pellucid glands, divided to c. 3/4 into 5 broadly-obovate, subequal lobes; abaxial surface of corolla partially tomentose, adaxial surface often ciliate near the throat, otherwise glabrous; tube of corolla ± infundibuliform, 1–2.7 mm of length, 1.5–2.9 mm of diameter; stamens 5, free, densely ciliate with white, clavate hairs which reach the connective of anthers; three posterior stamens 5–9.5 mm, two anterior stamens 7–12 mm; stamen filaments greenish-white, occasionally with a purple tinge; all five anthers reniform, mediofix, papillose on adaxial surface, 0.6–1.5 mm; style tomentose at the base, 6–10 mm, clavate at the apex; stigma hemispherical; capsule (excl. rostrum) 3.5–7.5 × 3–5.4 mm, ovoid to broadly ovoid, densely tomentose on early development, later glabrescent, with a rostrum 1–1.5 mm; seeds numerous, chestnut brown to dark brown, 0.7–1 × 0.5–0.7 mm, obpyramidal to ovoid-oblong, irregularly prismatic, faveolate with 3–7 pits in each longitudinal series.

#### Etymology.

Name derives from the genitive of the Latin word “cervus” in reference to the red deer of the National Park which were often observed in the subspecies habitat. The popular animals are a considerable threat to their own *Verbascum* as they consume the young inflorescences.

### Verbascum delphicum Boiss. & Heldr. subsp. delphicum var. filictorum

Taxon classificationPlantaeLamialesScrophulariaceae

Zografidis var. nov.

urn:lsid:ipni.org:names:77158534-1

**Figure 3.**
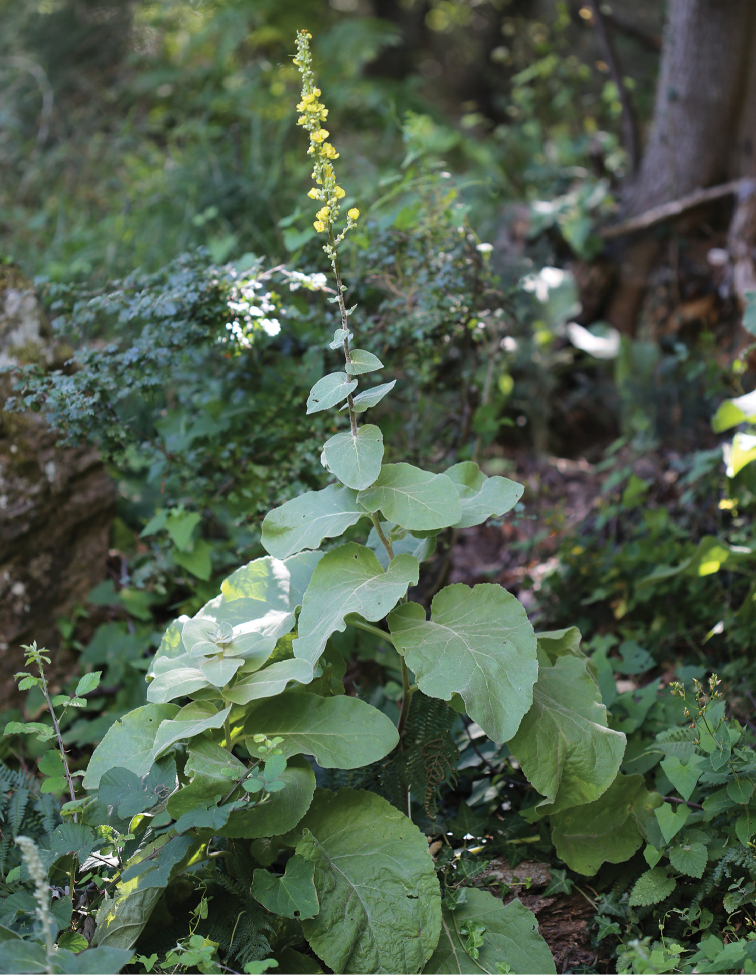
Verbascum delphicum subsp. delphicum var. filictorum Zografidis: Habit. A sterile stem is discernible next to the flowering stem.

Figure 3 ; Suppl. material 2.

#### Diagnosis.

Variety *filictorum* differs from typical subsp. delphicum in that it produces sterile stems up to c. 80 cm of height instead of basal leaf-rosettes.

#### Type.

GREECE. Ins. Evia. Above the settlement of Ag. Dimitrios, 38°06'N, 24°26'E, in patches of *Pteridium aquilinum*, 500 m, 4 July 2015, *A. Zografidis* 113. (Holotype: ATH, Isotype: ATHU)

#### Description.

Polycarpic eglandular- and minutely glandular-hairy short-lived perennial herb — or less often monocarpic biennial — producing a well-branched taproot and a sterile, erect or ascending, terete, leafy stem in the first year of vegetative growth, followed by the production of an additional sterile stem and a terete flowering-stem in each of the succeeding few years of vegetative and reproductive growth. Eglandular hairs dendritic, 0.4–2 mm of length, more or less covering the whole aerial part of the plant; glandular hairs minute, sparse, present on leaves, bracts, bracteols and calyx segments, visible by microscopy; fully developed first-season leaves whitish- to yellowish- and softly-tomentose above, grayish-white and softly tomentose beneath; sterile stems up to 80 cm in height; fertile stems 40–180 cm of height, green to reddish-black, glabrescent but ± persistently, tomentose below; lower cauline-leaves petiolate; petiole up to 15 cm of length; lamina ovate to widely ovate, 1.2–1.9 of length to width ratio, obtuse-truncate to cordate at the base, crenate, obtuse at apex; larger leaf laminas 16–40 × 11–24 cm; middle cauline-leaves similar but progressively smaller and often subacute at the apex, shortly petiolate; upper cauline-leaves small, sessile, ovate-cordate, subacute at the apex; all cauline leaves alternate; Inflorescence 25–60 cm of height, simple or sparingly branched at the base with short, sub-erect branches and then inflorescence narrowly pyramidal in outline; flowers arranged in clusters of pedicellate, compacted cymes (glomerules), ± crowded at least above, consisting of 3 – 12 flowers; bracts 3–7 × 2–4 mm, ovate to lanceolate, acute to acuminate or cuspidate, glabrescent; bracteoles present, similar to bracts but smaller; longer pedicels 4–10 mm of length, tomentose, ± glabrescent; calyx divided almost to the base into 5 lanceolate to lanceolate-linear, acute segments, 3.5–6 × 1.1–1.7 mm; abaxial surface of calyx tomentose, ± glabrescent; corolla rotate, flat, 1.5–2.8 cm of diameter, yellow, with or without indistinct purple marks on the throat, with pellucid glands, divided to c. 3/4 into 5 broadly-obovate, subequal lobes; abaxial surface of corolla partially tomentose, adaxial surface sometimes ciliate near the throat, otherwise glabrous; tube of corolla ± infundibuliform, 1–2.3 mm of length, 1.5–2.2 mm of diameter; stamens 5, free, ciliate with white, clavate hairs which reach the connective of all anthers or do not reach the connective of the anterior stamens; three posterior stamens 5–8 mm, two anterior stamens 6–10 mm; stamen filaments orange; all five anthers reniform, mediofix, papillose on adaxial surface, or glabrous on adaxial surface of the connective of anterior stamens, 0.8–1.4 mm; style tomentose at the base, 6–9 mm, slightly clavate at the apex; stigma hemispherical; capsule (excl. rostrum) 3.5–7 × 3–5 mm, ovoid to broadly ovoid, densely tomentose on early development, later glabrescent, with a rostrum 1–1.5 mm; seeds numerous, chestnut brown to dark brown, 0.7–1.1 × 0.5–0.7 mm, obpyramidal to ovoid-oblong, irregularly prismatic, faveolate with 4–8 pits in each longitudinal series.

#### Etymology.

Name is in reference to the often abounding in ferns habitat of the variety.

#### *Verbascum delphicum* specimens examined.

subsp. cervi: GREECE. Attica: In regione abietina montis Parnethis, 5–18 June 1911, Tuntas s.n. (GB)

subsp. delphicum: GREECE. Ins. Evvia: In rupidus ad cacumen m. Delphi, 5000’, 19 August 1948, Heldreich, 2040 (G-BOISS); In regione sylvatica & superiore montis Dirphyis (Delphi hod.) usque ad cacumen, 3000’-5500’ 31 July–5 August 1858, Heldreich 799 (K, P); Insula Evvia meridionalis, Montes Ocha, in valle infra Hagios Dimitrios, ca. 600 m, 21 May 1955, K. H. Rechinger, 17181 (W); Insula Evvia meridionalis, Montes Ocha, in querceto-castanetis vers us Kallianou, ca. 600 m, 22 May 1955, K. H. Rechinger, 17220 (W); Insula Evvia meridionalis, 3 km a promontorio Kaphireos occidentem versus, 22 June 1958, K. H. Rechinger, 18953 (W).

## Results and discussion

The differences between Verbascum delphicum subsp. cervi and subsp. delphicum are presented in Table 1, whereas a comparative illustration is presented in Figure 2. A three-year field survey of both insular and mainland populations and examination of cultivated plants confirmed the constancy of the distinctive features. Subspecies cervi is characterized by its smaller and more elongated leaves with the base of leaf-laminas obtuse to long-cuneate — vs. ± truncate or even cordate in subsp. delphicum — and by a shorter, harsher and darker indumentum. Another significant character is the color of the stamen filaments: either greenish-white in subsp. cervi or deep-orange in subsp. delphicum. I note that this character is almost lost with drying. Life-cycle is an additional interesting trait. In his Flora Orientalis, Boissier has cited *Verbascum delphicum* as a biennial herb (Boissier 1856), a vague conception which has been perpetuated in various floras ever since (Ferguson 1972; Raus 1986). Although biennial individuals are indeed found among its populations, the autonymous subspecies is more frequently a short-lived perennial herb as evidenced by the concomitant existence of the flowering stem and either of an additional leaf-rosette or of a previous-year withered infructenscence or of both on the same plants. On the other hand, subsp. cervi has clearly drifted towards the biennial life-cycle and very rarely do the plants exceed the usual two years lifespan — I have seen only two such individuals.

**Table 1.** Diagnostic characteristics of Verbascum delphicum subsp. cervi and subsp. delphicum. Numbers in brackets indicate mean and standard deviation values.

	Verbascum delphicum subsp. cervi	Verbascum delphicum subsp. delphicum
Life cycle	Monocarpic biennial, rarely polycarpic short-lived perennial	Polycarpic short-lived perennial or monocarpic biennial
Indumentum of first year mature rosettes on adaxial surface of leaves	Yellowish to brownish, harsh	Grayish-white to yellowish, soft
Indumentum of rosette leaves on abaxial surface	Grayish-white or yellowish to brownish, thin	Grayish-white, thick
Dendritic hairs (mm)	0.2–0.8	0.4–2
Length to width ratio of leaf-lamina (rosette leaves)	1.5–3.7 [2.5 ± 0.5]	1.2–1.9 [1.6 ± 0.2]
Lamina of larger leaves, (cm)	6–22 [14.7 ± 4.4] × 3–11 [6.2 ± 1.9]	16–40 [26.6 ± 7.1] ×11–24 [17 ± 3.5]
Base of lamina	Obtuse-cuneate	Obtuse-truncate to cordate
Stamen filaments	Greenish-white	Orange
Hairs on connective of anterior stamens (adaxial surface)	Present	Frequently absent

On Mt Parnitha and on the mountainous region of central Evvia — Mts Dirfi and Xirovouni — *Verbascum delphicum* inhabits Greek-fir or mixed *Abies*-*Castanea* woodlands, flowers from early-mid-June to early-mid-August and matures seeds from July to August. On Mt Ochi of southern Evvia the species is found in open *Castanea* woodlands — *Abies* is absent from southern Evvia — or in sub-montate to montane, open shrublands dominated by thick patches of *Pteridium aquilinum* ferns. In these habitats of Mt Ochi, I have found that subsp. delphicum frequently produce sterile stems instead of basal leaf rosettes; the unusual trait gives a quite distinctive appearance to the plants but apart from it they are indistinguishable from typical individuals that grow in proximity. I propose this taxon to be classified as a new variety of subsp. delphicum (Figure 3 and Suppl. material 2). The variety might have evolved from the competition for sunlight inside the dense patches of ferns of these habitats, whereas I have never found it in central Evvia — not even in the shadier spots where subsp. delphicum produces the typical basal rosettes.

**Figure 4.**
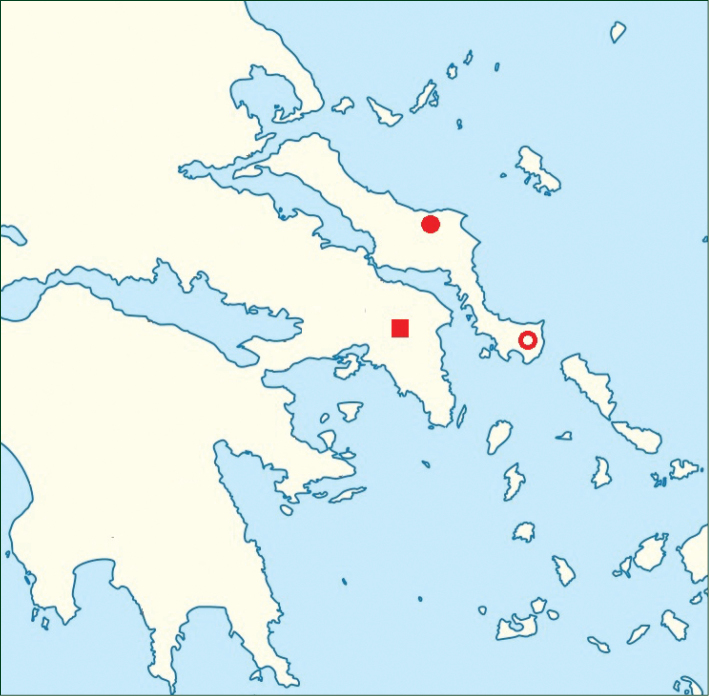
Distribution map of *Verbascum delphicum*: ■ subsp. cervi; ●, ○ subsp. delphicum; ○ var. filictorum.

Murbeck’s assumption that typical *Verbascum delphicum* existed on Mt Parnitha was based on the following material he cited in his monograph: i) a collection by Heldreich — “*Parnes, reg. abiet. occid., leg. HELDREICH 1854, n. 2902 [Hb. Berl.]*” — even though Heldreich himself never reported *Verbascum delphicum* from mainland Greece and the date of the collection, which has unfortunately been destroyed during World War II (Th. Raus, personal communication, 27 August 2015), precedes by two years that of the original description of the species; ii) three collections by collector Bassilios Tuntas — “*Parnes, reg. abiet., occid., leg. TUNTAS 5/7/1911, n. 1247 [Hb. Hal.; Hayek].- Mt Parnes, prope cacumen, leg. TUNTAS 21/7/1907, n. 356 [Hb. Hal.]; 13/6/1909, n. 810 [Hb. Hal.]*” — which, however, were not found either in WU-Generale, WU-Halácsy-Graecum or WU-Halácsy-Europaeum (W. Till, personal communication, 15 March 2016). Perhaps the only surviving voucher is kept in the Gothenburg Herbarium and likely corresponds to the aforementioned n. 1247 specimen of the collector, although the serial number is missing. Nevertheless, on the sheet there is a label with Murbeck’s name and handwriting that states “*Verbascum delphicum*”. Having seen the specimen I have no doubt it represents subsp. cervi on account of the characteristic small basal leaves with an obtuse-cuneate base and the short indumentum. It is interesting to note that the aforementioned specimen includes the whole aerial part of a plant arranged on a single sheet, which is actually feasible for a medium individual of subsp. cervi — e.g., see the Holotype, Suppl. material 1. On the contrary, a medium individual of subsp. delphicum is impossible to completely fit on a voucher sheet because the basal leaves are too big. I conclude that on Mt Parnitha there exists a single infraspecific taxon of *Verbascum delphicum*, namely subsp. cervi, allopatric to the insular subsp. delphicum which is confined to Evvia. The species had not been collected from the mainland for almost a century, but its existence was confirmed by a recent floristic study on the mountain (Aplada et al. 2007), (E. Aplada, personal communication 1 September 2016). Other reports of *Verbascum delphicum* from the mainland — from the Peloponnese cited in (Dimopoulos et al. 2013) or from Mt Pendeli cited in (Vladimirov et al. 2012) — are in error (Th. Raus, and K. Polymenakos, personal communications respectively). The distribution map of *Verbascum delphicum* is presented in Figure 4.

### Suggested conservational status

Verbascum delphicum subsp. cervi is restricted to the core of the National Park of Mt Parnitha where it apparently constitutes a single, fragmented population within an area of occupancy of less than 1 km^2^ and a total population size of no more than 50 mature individuals (August 2016). The alarmingly rare new taxon has suffered habitat loss with the devastating fire of 2007 on the mountain and is now under direct threat by the overpopulated deer of the National Park which consume the developing inflorescences; the bitten plants respond by producing a few new small flowering shoots but seed yield is expected to be severely affected. Importantly, the population size of Verbascum delphicum subsp. cervi has been reduced about 20% over the last three years and the taxon is apparently critically endangered according to IUCN Red list criteria C(2a(i,ii)) and D(1) (IUCN 2001).

### Comments on taxonomic affinities and phytogeography

Considering the recent radiation in the genus *Verbascum* (Ghahremaninejad et al. 2014) and the strong phytogeographic connection between Ins. Evvia and Sterea Ellas (Trigas 2003), it is reasonable to assume that Verbascum delphicum subsp. cervi represents a neoendemic taxon that has diverged from the insular subspecies through the process of allopatric subspeciation. On the other hand, the infrageneric relationships of *Verbascum delphicum* are more difficult to infer. In particular, Murbeck places *Verbascum delphicum* under Sect. Bothrosperma Murb., Subsect. Fasciculata Murb., B. *Isandra* Franch., p. p., a. *Bracteolata* Murb., α *Umbellulifera* Murb., 1. *Adenanthera* Murb. and further groups it with the widespread in the Balkans *Verbascum banaticum* Schrad. on account of the white stamen-hairs and the relatively long pedicels. However, the two species are very different and only doubtfully closely related — *Verbascum banaticum* is an eglandular herb with lobed and pinnatisect at the base of the lamina rosette-leaves, freely branched, leafy, lax inflorescences and has smaller all flower parts and capsule. Rather I suspect that *Adenanthera* — i.e. having papillate connective of the anthers of the anterior stamens — is a non-monophyletic trait and that *Verbascum delphicum* is actually more closely related to some *Leianthera* Murb. — i.e. with glabrous connective — species of the centered but not restricted to the Balkans group of *Verbascum nigrum* L., *Verbascum chaixii* Vill., *Verbascum lanatum* Schrad. and *Verbascum glabratrum* Friv. These species show considerable resemblance to *Verbascum delphicum* with respect to general leaf morphology, indumentum and habit. Moreover, additionally to their glabrous connective of anterior stamens, these species all differ from *Verbascum delphicum* in the purple stamen-hairs and in the more or less striate stems. I should also note at this point that the connective of the anterior stamens of *Verbascum delphicum* is often glabrous too and this is particularly evident for subsp. delphicum — Figure 2F shows one such stamen with the hairs not found on the connective. Intriguingly, *Verbascum delphicum* also shows considerable phenetic resemblance with two Asiatic species, namely *Verbascum discolor* Murb. from the Amanus Mountains and *Verbascum antitauricum* Hub.-Mor. from the Anti-Taurus mountains, both of south-central Turkey. *Verbascum discolor* is generally a more glabrous plant than *Verbascum delphicum* with glabrous connective of anterior stamens, glabrous outer surface of the corolla and a more branched and laxer inflorescence. *Verbascum antitauricum*, similarly to *Verbascum delphicum* has either papillate or glabrous connective of anterior stamens but has bicolored stamen-hairs, glabrous corollas on the outer surface, more or less shorter pedicels and it is eglandular. In any case, the differences between *Verbascum delphicum* and the Turkish species are not more pronounced than those between *Verbascum delphicum* and the aforementioned Balkan species — compared individually — and thus additional data from molecular phylogenetics and/or cytological studies are needed for clarifying the topic of *Verbascum delphicum* infrageneric affinities.

## Conclusions

Both infraspecific taxa of *Verbascum delphicum* described in this study are distinct and easily recognized, whereas the species closest relative remains unknown. Subspecies cervi is apparently a critically endangered taxon.

## Supplementary Material

XML Treatment for Verbascum delphicum Boiss. & Heldr. subsp. cervi

XML Treatment for Verbascum delphicum Boiss. & Heldr. subsp. delphicum var. filictorum

## Supplementary materials

Supplementary material 1
Verbascum delphicum subsp. cervi Zografidis subsp. nov., holotype. Data type: JPEG file This dataset is made available under the Open Database License (http://opendatacommons.org/licenses/odbl/1.0/). The Open Database License (ODbL) is a license agreement intended to allow users to freely share, modify, and use this Dataset while maintaining this same freedom for others, provided that the original source and author(s) are credited.Aris Zografidis

Supplementary material 2
Verbascum delphicum subsp. delphicum var. filictorum Zografidis var. nov., holotype. Data type: JPEG file This dataset is made available under the Open Database License (http://opendatacommons.org/licenses/odbl/1.0/). The Open Database License (ODbL) is a license agreement intended to allow users to freely share, modify, and use this Dataset while maintaining this same freedom for others, provided that the original source and author(s) are credited.Aris Zografidis
