# Supplementary figures and images for: Two new infraspecific taxa of Verbascum delphicum (Scrophulariaceae, Scrophularieae) from mainland Greece and the island of Evvia
Source: PhytoKeys. 2016 Nov 10;(74):107–22. doi: 10.3897/phytokeys.74.10381 (PMC5234551; doi:10.3897/phytokeys.74.10381)

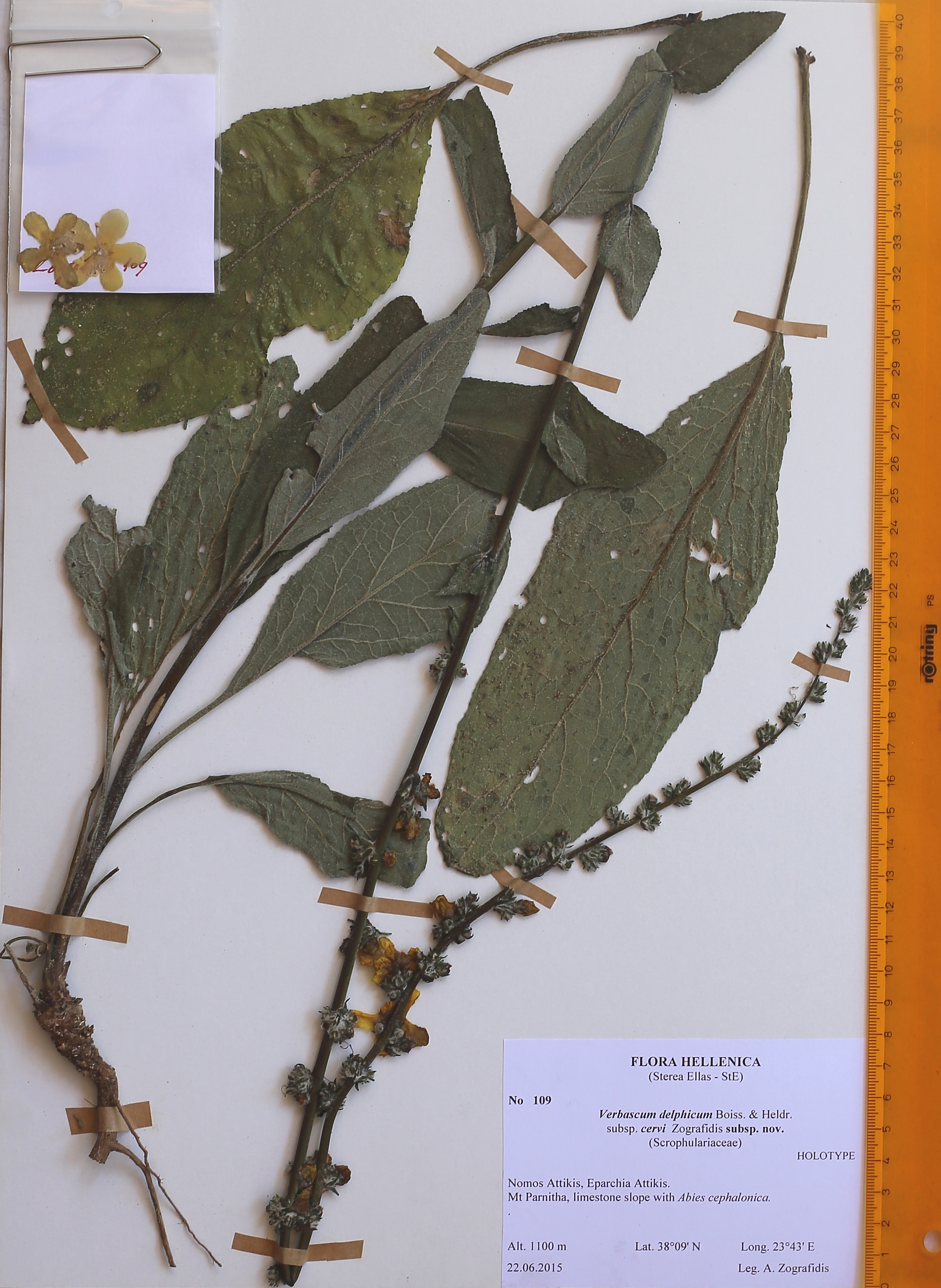

Supplement: Supplementary material 1 — Verbascum delphicum subsp. cervi Zografidis subsp. nov., holotype. [file phytokeys-074-107-s001.jpg]

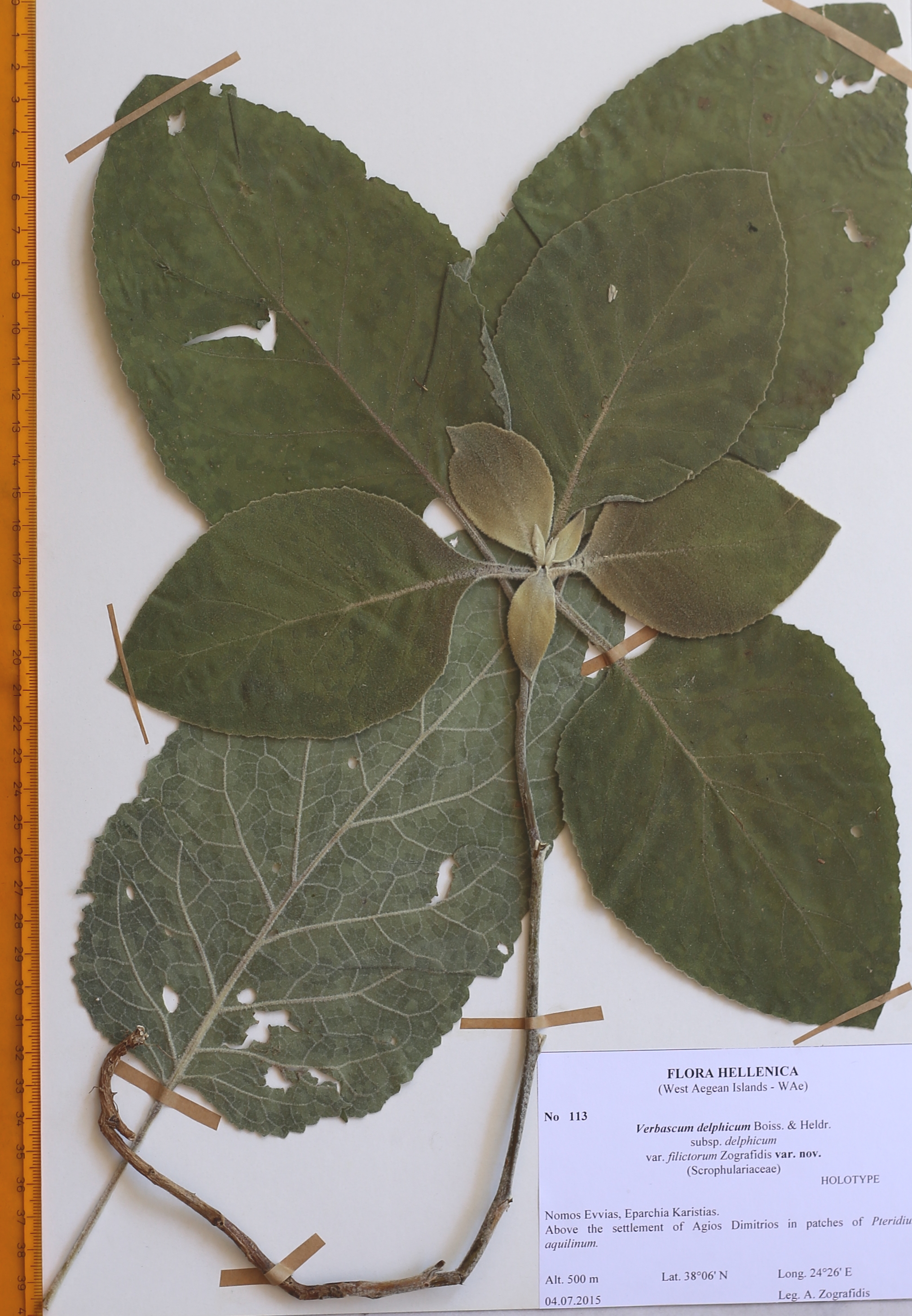

Supplement: Supplementary material 2 — Verbascum delphicum subsp. delphicum var. filictorum Zografidis var. nov., holotype. [file phytokeys-074-107-s002.jpg]
